# The value of D-dimer in the detection of early deep-vein thrombosis after total knee arthroplasty in Asian patients: a cohort study

**DOI:** 10.1186/1477-9560-6-5

**Published:** 2008-05-28

**Authors:** Chung-Jen Chen, Ching-Jen Wang, Chung-Cheng Huang

**Affiliations:** 1 Department of Internal Medicine, Chang Gung Memorial Hospital-Kaohsiung Medical Center, Chang Gung University College of Medicine, Kaohsiung, Taiwan; 2 Department of Orthopedic Surgery, Chang Gung Memorial Hospital-Kaohsiung Medical Center, Chang Gung University College of Medicine, Kaohsiung, Taiwan; 3 Department of Diagnostic Radiology, Chang Gung Memorial Hospital-Kaohsiung Medical Center, Chang Gung University College of Medicine, Kaohsiung, Taiwan

## Abstract

**Background and purpose:**

The relationship of D-dimer and deep-vein thrombosis (DVT) after total knee arthroplasty (TKA) remains controversial. The purpose of this study was to assess the value of D-dimer in the detection of early DVT after TKA.

**Methods:**

The measurements of plasma D-dimer level were obtained preoperatively and at day 7 postoperatively in 78 patients undergoing TKA. Ascending venography was performed in 7 to 10 days after surgery. The plasma D-dimer levels were correlated statistically with the venographic DVT.

**Results:**

Venographic DVT was identified in 40% of patients. High plasma D-dimer level >2.0 μg/ml was found in 68% of patients with DVT and 45% without DVT (P < 0.05). Therefore, high D-dimer level greater than 2.0 μg/ml showed 68% sensitivity, 55% specificity, 60% accuracy, 50% positive predictive rate and 72% negative predictive rate in the detection of early DVT after TKA.

**Conclusion:**

High plasma D-dimer level is a moderately sensitive, but less specific marker in the detection of early of DVT after TKA. Measurement of serum D-dimer alone is not accurate enough to detect DVT after TKA. Venography is recommended in patients with elevated D-dimer and clinically suspected but asymptomatic DVT after TKA.

## Background

Recent studies have shown that the incidence of deep-vein thrombosis (DVT) after total knee arthroplasty (TKA) in Asian patients is as high as that of the Western countries [1-9]. Pharmaceutical prophylaxis significantly lowered the incidence of DVT, but none of currently available modalities showed total prevention of DVT [3]. Deep-vein thrombosis after TKA is sometimes difficult to diagnose because more than half of DVT cases after TKA are asymptomatic [3], which might cause propagation of the clots leading to pulmonary embolism [7]. Therefore, the effective management of DVT relies on the early detection of DVT. Venographic study is considered the gold standard in the diagnosis of DVT of the lower extremity. Venography, however, is an invasive procedure that can incur certain risks and is expensive [3]. As a result, patients often refuse venography and orthopedic surgeons thus are reluctant to recommend the procedure. Ultrasonography is a reasonable alternative, but the sensitivity of ultrasound in detecting calf DVT is inferior to venography and the examination is also expensive and time consuming [10,11]

Many studies of non-traumatized patients reported that the cross-linked fibrin degradation by-product associated with elevated D-dimer level is indicative of the presence of a clot, including DVT [8,12-26]. The measurement of D-dimer is simple, fast, convenient and inexpensive, and has the economic potential of reducing the use of more complex and expensive tests such as venography and ultrasonography. However, the interpretation of the D-dimer test in cases with DVT after TKA is still controversial [27-33]. The purpose of this prospective study was to evaluate the value of plasma D-dimer level in the detection of early DVT after TKA in Asian patients.

## Methods

The Institutional Review Board of our hospital approved this study. All patients signed an informed consent. From January 2002 to June 2002, 89 consecutive patients undergoing TKA were enrolled in this study. Eleven patients were excluded because of incomplete data, only those taking D-dimer tests both prior to surgery and after surgery were enrolled. The remaining 78 patients were included in the final analysis. There were 64 females and 14 males with an average age of 67.3 ± 8.6 years (range 34 to 85). The mean body height was 151.3 ± 7.5 cm (range 130 to 172), and the mean body weight was 67.0 ± 12.1 kg (range 47 to 110). The right knee was affected in 36 cases and the left knee in 42. None of the patients had a family history of DVT. Routine preoperative work-ups included history and physical, complete blood count, coagulation profiles, chemistry profiles, electrocardiogram, and x-rays of the chest and knee. All operations were performed under either general or spinal anesthesia. Only one type of prosthesis (Advantim, Wright Medical, TN, USA) was used and all components were cemented. Prophylactic antibiotic (cefamezine) was given for 24 hours, but no pharmaceutical DVT prophylaxis was used because that pharmaceutical prohylaxis was not routinely used in most hospitals in our country. Therefore, we could study the natural occurrence of DVT and assess whether or not D-dimer can predict DVT development. All patients received the same protocol of postoperative management, including physical therapy with partial weight bearing on the operated leg, muscle exercise and continuous passive motion (CPM) starting on the second postoperative day. Patients were discharged from the hospital when they were independent on ambulation with a walking aid, and continued outpatient physiotherapy until full recovery.

Blood samples for plasma D-dimer measurement were drawn on the day before surgery and the 7^th ^day after surgery. The measurements of plasma D-dimer level were performed with the D-dimer plus (DADE BEHRING, Marburg, Germany) by microlatex assay, which using a monoclonal antibody to detect only cross-linked D-dimer fragments. The total range of D-dimer measurements extended from 0.05 to 6.5 μg/ml. The D-dimer levels were defined as negative (< 0.25 μg/ml), minimal (0.25 – 0.5 μg/ml), low (0.5 – 1.0 μg/ml), medium (1.0 – 2.0 μg/ml) and high (> 2.0 μg/ml). Ascending venography was performed in 7 to 10 days postoperatively. The venographies were interpreted for DVT by two radiologists blinded to the nature of the study.

### Statistical analysis

The data of D-dimer levels were statistically correlated with the occurrence of venographic DVT using Chi Square test with statistical significance set at P < 0.05.

## Results

### The prevalence of DVT

DVT was identified in 31 of 78 patients resulting in an incidence of 40%. There were 30 (97%) distal and one proximal DVT (3%). There was no pulmonary embolism. Symptomatic DVT including pain and swelling of the leg or skin discoloration and calf or thigh girth enlargement was observed in 9 patients (29%) with positive venographic DVT. The remaining 22 cases (71%) were asymptomatic.

### D-dimer level

**Figure 1:**
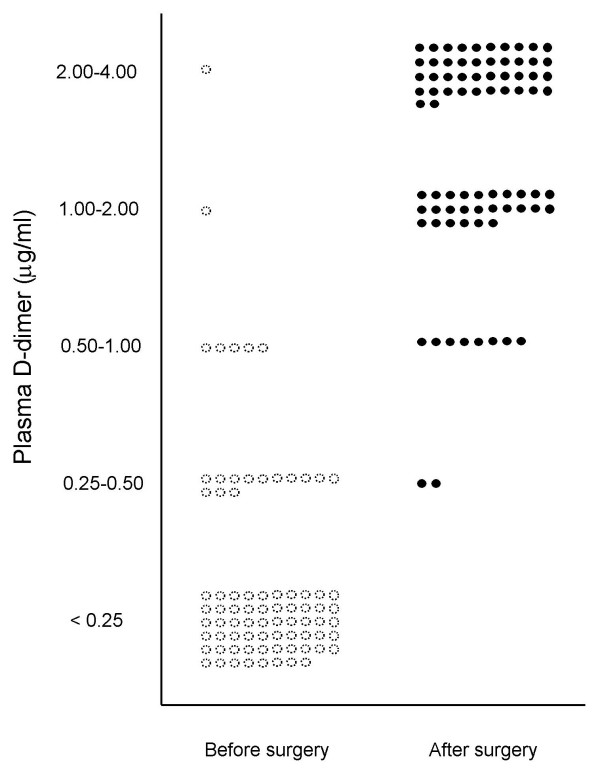
The plasma D-dimer levels before and after total knee arthroplasty.

**Figure 2:**
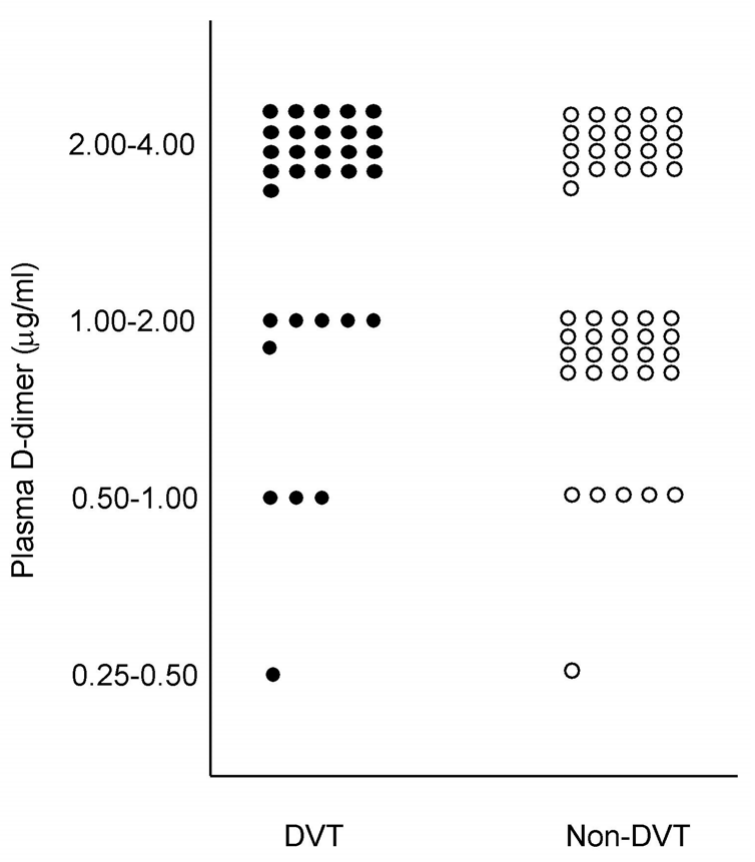
The plasma D-dimer levels in patients with and without deep-vein thrombosis after total knee arthroplasty.

The results of plasma D-dimer level before and after surgery are shown in Fig. 1. Before surgery, 20 of 78 patients (26%) showed positive D-dimer reaction, although high D-dimer level of greater than 2.0 μg/ml was noted in only one patient (1.3%). After surgery, all 78 patients showed positive D-dimer reaction including 43 patients (55%) with D-dimer level of greater than 2.0 μg/ml and 35 patients (45%) with lower D-dimer level of less than 2.0 μg/ml. The plasma D-dimer levels in patients with and without DVT are shown in Fig. 2. Thirty-one patients (40%) developed DVT postoperatively whereas 47 patients did not.

### D-dimer level and DVT

The correlations of D-dimer level and the occurrence of venographic DVT are shown in Table 1. High plasma D-dimer level of greater than 2.0 μg/ml was noted in 68% (21 of 31) of patients with positive DVT versus 45% (21 of 47) of patients without DVT (P < 0.05). Lower plasma D-dimer level (less than 2.0 ug/ml) was seen in 32% (10 of 31) of patients with DVT and 55% (26 of 47) of patients without DVT.

**Table 1:** Sensitivity and specificity of different D-dimer levels in DVT after TKA

D-dimer level	DVT N = 31	Non-DVT N = 47	Sensitivity %	Specificity %
>0.25 ug/ml VS <0.25 ug/ml	31 VS 0	47 VS 0	100	0
>0.5 ug/ml VS <0.5 ug/ml	30 VS 1	46 VS 1	97	2
>1 ug/ml VS <1 ug/ml	27 VS 4	41 VS 6	87	13
>2 ug/ml VS <2 ug/ml	21 VS 10	21 VS 26	68	55

### The sensitivity and specificity of D-dimer level in DVT

The calculations of sensitivity, specificity, accuracy, positive and negative predictive values of plasma D-dimer level in the detection of early DVT are shown in Table 1. When high plasma D-dimer level (> 2.0 μg/ml) is used as a biological marker for early DVT after TKA, sensitivity is 68%, specificity is 55%, accuracy is 60%, positive predictive rate is 50% and negative predictive rate is 72%.

## Discussion

Clinical diagnosis of DVT is unreliable. In symptomatic DVT, compression ultrasound is very accurate [34]. However, patients with asymptomatic DVT frequently showed normal ultra sonography [13,22]. Ascending venography is the most reliable method in the detection of DVT of the lower leg, but it is expensive, time consuming and incurs certain risks [3]. The D-dimer test might be an attractive alternative in early detection of DVT. Many studies of non-traumatized patients reported that the plasma D-dimer assay is indicative of DVT. However, the value of D-dimer in DVT after TKA remains controversial. Shiota el al reported that a high level of D-dimer on the 7^th ^postoperative day was most sensitive with 95.5% for THA and 94.4% for TKA and most specific with 96.9% for THA and 90.0% for TKA in the diagnosis of DVT after THA and TKA, and concluded that high level of D-dimer on postoperative day 7 [27]. Arnesen et al reported a correlation of D-dimer with late occurring DVT at day 35 after hip replacement surgery [28]. On the contrary, other studies reported a negative value of D-dimer in the diagnosis of DVT after TKA [29-33]. Bounameaux et al [29] reported that measurement of plasma D-dimer concentration is of no value for predicting, diagnosing or ruling out DVT in patients undergoing total knee arthroplasty. Harper et al [30] stated that the SimpliRED assay in D-dimer measurement is too insensitive to use as a reliable exclusion test in cases of suspected DVT.

The results of the current study showed that high plasma D-dimer level of higher than 2.0 μg/ml at day 7 postoperatively is moderately sensitive, but not necessarily specific enough in the detection of early DVT after TKA. The findings of the current study are in agreement with those of the European reports, but different from the findings of Japanese study (Table 2). The results of the study suggested that D-dimer test alone is not accurate enough in the detection of early DVT because the plasma D-dimer level can be influenced by comorbid conditions such as cancer, infection and surgery [14].

**Table 2:** Comparison of D-dimer and DVT after TKA among different studies

Place	Author	N	Sensitivity%	Specificity%	reference
Japan	Shiota et al	28	94	90	[27]
Switzland	Bounameaux	118	73	37	[29]
France	De Prost	11	100	0	[32]
France	Abraham	188	57	71	[33]
Taiwan	Chen et al	78	68	55	present study

## Conclusion

Measurement of plasma D-dimer level is not accurate enough in detecting DVT after TKA. Venography is recommended in patients with elevated D-dimer and clinically suspected but asymptomatic DVT after TKA.

## Competing interests

The authors declare that they have no competing interests.

## Authors' contributions

CJC participated in the study with the analysis of data and review of the references.

CJW participated in the study with contribution of the study design and manuscript preparation.

CCH participated in the study with interpretation and analysis of venography.

All authors read and approved the final manuscript.
